# Why and How Meet n-3 PUFA Dietary Recommendations?

**DOI:** 10.1155/2011/364040

**Published:** 2010-12-08

**Authors:** Olivier Molendi-Coste, Vanessa Legry, Isabelle A. Leclercq

**Affiliations:** Laboratory of Hepato-Gastroenterology, Institut de Recherche Expérimentale et Clinique, Université Catholique de Louvain (UCL), GAEN 53/79 Avenue Mounier, 53, 1200 Brussels, Belgium

## Abstract

Obesity and the metabolic syndrome are systemic inflammatory diseases reaching epidemic proportions. Contemporary changes in human nutrition occurred characterized by increased consumption of fat and of vegetable oils rich in n-6 polyunsaturated fatty acids (PUFAs) together with decrease in n-3 PUFA-rich foods, resulting in an n-6/n-3 ratio of 10–20/1 in Western diet for a ratio around 1/1 in the diet of our ancestors. The literature provides compelling evidence for the health benefit of n-3 PUFA consumption on inflammation and metabolic syndrome prevention and treatment. Such evidence led to the establishment of comprehensive recommendations. However, we show here that, both in collective catering proposed to children and in hospital diet, it is not straightforward to meet such recommendations. Willingness of governments to institute changes, with accountable decisions on catering, nutritional education, and food processing, is required to face our neglected responsibility in promoting balanced diet and consumption of foods rich in essential nutrients in the general population.

## 1. Introduction

**Figure 1:**
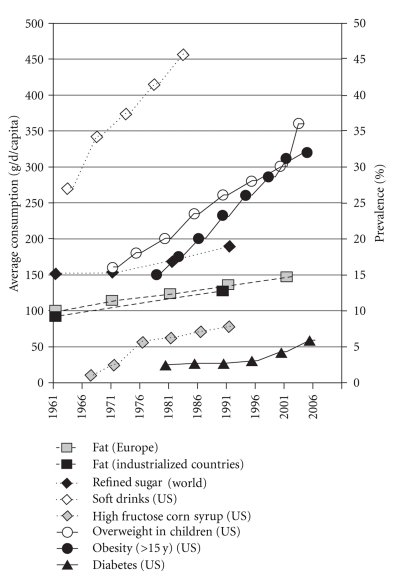
Evolution of Occidental dietary content in specific macronutrients and prevalence of obesity and diabetes in United States. Compiled from [11–13]; FAO, AGROSTAT.PC, 1993; Centers for Disease Control and Prevention, National Center for Health Statistics, Division of Health Interview Statistics, data from the National Health Interview Survey; French National Epidemiological Study on Overweight and Obesity: ObEpi-Roche 2009.

The metabolic syndrome (MetS) is defined as a cluster of symptoms such as visceral obesity, insulin resistance, elevated blood pressure, and dyslipidemia, associated with increased risk of type 2 diabetes, cardiovascular disease [1], nonalcoholic fatty liver diseases (NAFLD) [2], and some types of cancers [3]. This pathological condition is currently reaching epidemic proportions (Figure 1) and may soon represent the first health issue worldwide in terms of costs and mortality, even in developing countries. Although multifactorial processes participating are yet to be unraveled, there is a general agreement that the rising prevalence of MetS is largely due to the increasing incidence of adiposity [4]. Obesity, and in particular abdominal obesity or visceral fat, as well as the MetS has been identified as low-grade and systemic inflammatory conditions [5, 6], with an imbalance between pro- and anti-inflammatory molecules and elevated serum markers of inflammation [7]. Increased macrophage infiltration in adipose tissue and possibly liver [8, 9], as well as recruitment of lymphocytes [10] is recognized causes of inflammation and insulin resistance in this context. 

**Figure 2:**
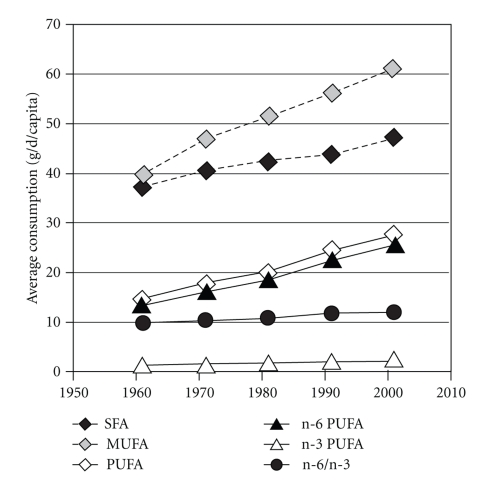
Evolution of fatty acids consumption in European Union. Adapted from [13].

During the past 15 years, research efforts have focused on the primary factors responsible for the MetS and its increasing prevalence worldwide. Contemporary to the rise in MetS prevalence, important changes in human nutrition and dietary habits were observed (Figures 1 and 2) in parallel with the adoption of a more sedentary life style, owing to industrialization and drastic changes in strategies for communication, crystallized under the emblematic so-called “occidental way of life”. Consequently, this has resulted in a disruption of the balance between energy intake and consumption/expenditure, as well as relative excess/deficiency in some metabolically relevant nutrients. Such modifications are thought to be part of the MetS epidemic.

In particular, a steady increase in dietary refined sugar and fructose, that parallels the rise in obesity and diabetes, is observed since the 70s due to the increased consumption of soda, soft drinks, and manufactured candy and pastry [11] and use of enriched (high) fructose corn syrup as sweetening agent (Figure 1) [11, 12, 14]. Concomitantly, the rate of fatty acid (FA) consumption has increased. Indeed, in today's diet, FA represents 28%–42% of total energy consumed by European populations [15], affording 128 g/d in developed countries in 1990, while in 1961 it was estimated to 93 g/d [16]. In ancestral nutrition, FA consumption was approximated around 20%–30% of energy intake [17, 18]. Also, qualitative changes in the type of FA taken in have occurred over the past 50 years, as depicted in Figure 2 for European populations [13]. These changes are characterized by increased consumption of saturated fat (especially from meat), vegetable oils rich in linoleic acid (LA, n-6 PUFA) on the one hand, and an overall decrease in n-3 PUFA intakes relative to n-6 PUFA on the other hand [19]. This was mainly attributable to insufficient consumption of fatty fish [20], reduced nuts, seeds, and whole-grain cereals in alimentation [21] and a progressive preferential use of safflower oil, poor in n-3 PUFA. Importantly, marine fish and especially fatty ones are the most important source of n-3 PUFA in the Occident. Aside from marine alga and newly engineered oils and supplements [20, 22], marine fish represent the only natural edible source of long-chain (LC) n-3 PUFA eicosapentaenoic and docosahexaenoic acids (EPA and DHA), the most biologically active n-3 PUFA [23]. In Occidentalized countries, fish consumption is very variable and globally low [24, 25]. Intensive farming could also be an additional factor contributing to insufficient n-3 PUFA consumption as n-3 PUFA content in some species of farmed fish, as rainbow trout [26], bream [27], salmon coho, or catfish [28], are reduced compared to their wild counterparts. As a result, n-6 PUFA consumption has become progressively much higher than that of n-3 PUFA [29], so that Western diets have a n-6/n-3 ratio ranging from 10/1 to 20/1 for a ratio of 1/1 in the diet of our ancestors [17, 30].

## 2. Metabolic Consequences of Altered Fatty Acid Nutritional Intakes

A body of epidemiological evidence highlights that high consumption of saturated FAs and trans-FAs may have adverse effects on lipid and glucose homeostasis and evolution towards the MetS [31]. The mechanisms involved are (i) the accumulation of toxic diacylglycerol and ceramides, (ii) the activation of nuclear factor-*κ*B, protein kinase C, and mitogen-activated protein kinases which induce the expression of inflammatory genes in adipose tissue and immune cells, (iii) the decrease of peroxisome proliferator-activated receptor (PPAR) *α* and adiponectin levels and consequent decreased oxidation of FA and glucose, and (iv) recruitment of immune cells in adipose tissue and muscle [32]. 

The recent literature provides convincing evidence of the detrimental role of low dietary n-3 PUFA for the MetS and the cardiovascular risk. It has been shown that n-3 PUFA in muscle membrane phospholipids are inversely related to insulin resistance, whereas the amount of LA (n-6 PUFA) incorporation into membrane phospholipids is positively related to insulin resistance. The links between n-3 PUFA and MetS is confirmed in many studies conducted independently around the world. For example, in The Multiple Risk Factor Intervention Trial involving 6,250 middle-aged American men determined to be at high risk of coronary heart diseases, evaluation using four annual dietary recall interviews showed that low n-3 PUFA consumption was associated with increased mortality. On the contrary, no significant association with mortality was detected for LA (n-6), which was the predominant dietary PUFA [33]. A study by Delavar et al. involving 984 random sampled Iranian women (30–50 y) suggests that a diet that lacks n-3 PUFA and vitamins-rich foodstuffs such as fish, vegetables, and nuts, increases the likelihood of having MetS [34]. Similarly, in France, low consumption of fish, and thus of n-3 PUFA, is associated with a higher probability of MetS, as assessed on 912 men (45–64 y) [21]. 

Distinct beneficial effects of fish (LC n-3 PUFA) consumption have been reported on insulin sensitivity, type 2 diabetes mellitus (T2DM), lipid profile, and risk for death from coronary heart diseases in healthy individuals [35–38] or of *α*-linolenic acid (ALA) intake on reduced risk of myocardial infarction [39]. Total n-3 PUFA supplies were also associated with higher levels of anti-inflammatory markers (soluble IL-6r, IL-10, TGF*β*) in healthy adults [40]. Together, all those observations support that dietary n-3 PUFA may specifically influence the development of insulin resistance and progression of the MetS, and associated cardiovascular risk.

## 3. Metabolism of n-3 PUFA

**Figure 3:**
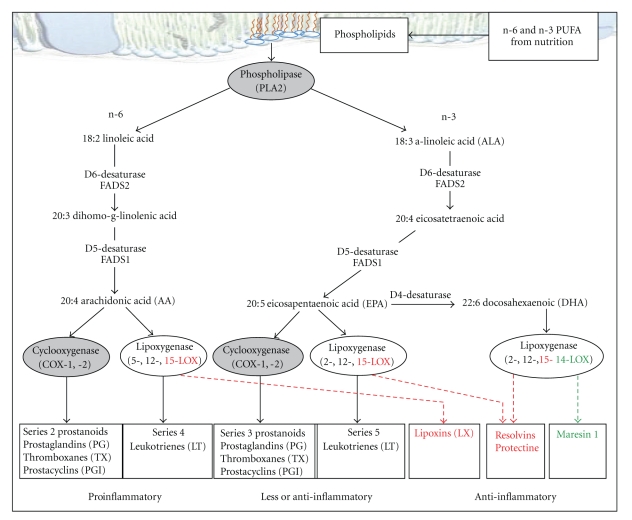
Metabolism of polyunsaturated fatty acids and their signaling molecules. Adapted from [41–43]. The 3D plasma layer image was modified from the one provided by Mélanie Villeneuve, La cellule animale, CCDMD, 2008 (http://www.ccdmd.qc.ca/ri/cellule/index.php?nh=18).

FAs, whether saturated, mono- (MUFA) or polyunsaturated, are oxidized in the mitochondria and represent the most energetic substrates of the diet. They are incorporated into phospholipids as the major components of cellular membranes or packaged into triglycerides for storage and export. The essential PUFA of the n-3 series (EPA and DHA, found in fish oil and ALA, precursor of EPA and DHA, found in nut, soy, and rapeseed oils) and of the n-6 series (arachidonic acid -AA- and LA found in sunflower and nut oils) are precursors for different signaling molecules. Initial steps in their metabolism are desaturations catalyzed by rate-limiting Δ6 and Δ5 desaturases (Figure 3). In humans, Δ5 and Δ6 desaturase activities, and thereby the conversion rate of ALA to EPA/DHA, are low and can further be modulated by genetic and epigenetic factors and dietary co-factors, including magnesium, zinc, and vitamin B6 [44, 45]. Therefore, exogenous sources of EPA/DHA are important as they generate the most potent n-3 PUFA-derived protective mediators [23].

Site-specific oxygenation by cyclooxygenases (COX) and lipoxygenases (LOX) produces different signaling molecules among which are eicosanoids, comprising prostaglandins (PGs), thromboxanes, and leukotrienes (LTs) (see Figure 3) [41–43]. While n-6 PUFA are substrates for synthesis of proinflammatory eicosanoids (series 2 prostanoids and series 4 LT), n-3 PUFA metabolism rather yields less or anti-inflammatory eicosanoids amongst them series 3 prostanoids and series 5 LT [46]. Some of these LC metabolites (EPA, DHA, PGI3, PGE1, and PGI2) may serve as endogenous inhibitors of the angiotensin converting enzyme and HMG-CoA reductase and as nitric oxide enhancers to produce antihypertensive, anti-inflammatory, and antiatherosclerotic effects by acting on vascular cells, leukocytes, and platelets [47] and function as signaling molecules via activation of peroxisome proliferator-activated receptor (PPAR) transcription factors regulating lipid metabolism [48]. There is also increasing evidence that n-3 PUFA directly protect against cellular aging and age-related diseases [49], possibly through reduction of telomeres shortening in leukocytes from patients with coronary heart disease [50]. Recently, classes of autacoids as the E- and D-series resolvins, protectins, and maresin 1 derived from LC n-3 PUFA as well as lipoxins derived from LC n-6 PUFA have been identified as specialized mediators that stimulate host defense and dampen inflammation, prevent platelet aggregation, lower blood pressure, have antiarrhythmic action, reduce LDL cholesterol, activate telomerase, and have cytoprotective properties [36, 43, 46]. Whereas some of the effects of PUFA are undoubtedly mediated by eicosanoids, the PUFA-mediated suppression of lipogenic and glycolytic genes is independent of eicosanoid synthesis and appears to involve a nuclear mechanism directly modified by PUFA [51]. Thus, n-3 PUFA mediate anti-inflammatory, anti-steatosis, and vascular protective effects through several mechanisms, including modifications in cell membrane composition and function, gene expression modulation, or distinct eicosanoids production. 

Importantly, genetic polymorphisms in Δ5 and Δ6 desaturase-encoding genes (FADS1 and FADS2) are associated with variation in n-6 and n-3 PUFA content in serum phospholipid fractions and tissues [52]. Also, polymorphisms altering activity of FADS1/2 [52], LOX5 [53], and COX2 [54] genes products have been related to increased deleterious effects of n-6 PUFA, which were blunted by increasing n-3 PUFA consumption. Acquired modifications in enzymatic machinery metabolizing PUFA have been described in association with obesity, insulin resistance, and cancer [48, 54]. High-energy diet, SFA, and transfats during perinatal period have been shown to repress the expression of Δ5 and Δ6 desaturases in both maternal and fetal tissues and influence PUFA metabolism in adulthood, representing an additional mechanism for decreased tissue and membrane LC n-3 PUFA [55]. Interestingly, relevant to the characterized consequences of diet for epigenetics [56], Devlin et al. reported hypermethylation of FADS 2 gene promoter associated to decreased Δ6 desaturase activity and DHA levels in the liver in a nutritional model of hyperhomocysteinemia [45]. 

This clearly underlies that, beside nutrition, genetic factors, concurrent pathological conditions, and epigenetic modifications also play an important role in the regulation of LC-PUFA metabolism and may thus influence the development of inflammatory and metabolic diseases. It also suggests interindividual variations for requirements in n-3 PUFA.

## 4. n-3 PUFA and Benefits in the Metabolic Syndrome

Next to epidemiological evidence, literature provides a profusion of data reporting that increased n-3 PUFA consumption in intervention studies may alleviate metabolic and cardiovascular risk. Thus, consistent with the inverse correlation found between fish and fish oil consumption and biomarkers of inflammation (TNF*α*, IL6, CRP) in many populations (healthy adults -[40, 57]; patients with insulin resistance [58]; coronary heart disease [59]; or the MetS [44, 60]), dietary enrichment in n-3 ALA and in EPA/DHA reduced low grade inflammation in at-risk populations [61–64]. 

Despite a relatively low accumulation in adipocytes [41], LC n-3 PUFA elicit beneficial effects on adipose tissue in obesity, as indicated by (i) reduced body fat mass and stimulated lipid oxidation [65], (ii) improvement of body weight and satiety regulation [66], (iii) amelioration of cytokines profile, including leptin and adiponectin [66], and (iv) reduction of inflammation [44, 60]. Additionally, n-3 PUFA have been shown to reduce adipose tissue macrophage infiltration associated with obesity in animal models [67], but this requires confirmation in humans. 

Human trials confirmed that LC n-3 PUFA from either fish or fish oil supplements as well as ALA enrichment significantly reduce blood triglyceride levels in patients with MetS in a dose-dependent manner [35, 68], an effect that appears to be mediated through inhibition of hormone-sensitive lipase and VLDL secretion, and increase in apo B liver degradation [35].

A body of evidence demonstrates that n-3 PUFA are involved in the control of glucose homeostasis and insulin sensitivity [69]. In murine models of obesity and insulin resistance, incorporation of LC n-3 PUFA into cell membrane phospholipids increases membrane fluidity and expression, affinity, and number of insulin receptors [58] as well as GLUT-4 protein level in adipocytes [70], thereby improving insulin sensitivity. In overweight patients, n-3 PUFA reduce transition from glucose intolerance to T2DM [29], and fish and fish oil consumption during energy reduction elicit an additional positive effects on insulin resistance [71]. However, a majority of n-3 PUFA administration trials did not prove efficient in reducing insulin resistance in T2DM [29].

Diet interventions with increased n-3 PUFA clearly demonstrated therapeutically, reliability in lowering mortality in subjects with cardiovascular diseases or the MetS [31, 72], an effect primarily related to increased DHA intakes [73]. This justifies the recommendation for daily consumption of 1 g/d of LC n-3 PUFA as part of secondary prevention strategy post ischemic heart event [74].

NAFLD, now recognized as the hepatic complication of the MetS, might trigger development of T2DM. Low dietary n-3 PUFA content induces hepatic desaturase activity [75]. In addition, enzymes involved in eicosanoid synthesis are located at the periphery of lipid droplets [76]. It is therefore plausible that in the context of diet- or obesity-induced fatty liver associated with excessive n-6/n-3 ratio, hepatic eicosanoid production is tilted towards proinflammatory components and participates to proinflammatory and insulin resistant status aggravating the MetS. Animal diet-induced obesity experiments clearly show that EPA and DHA supplementation reduces severity of NAFLD, if not preventing it [23], suggesting that increasing n-3 PUFA intake and fish consumption might prevent the occurrence of NAFLD in humans [2]. Properly conducted clinical trials are awaited to confirm this.

As important, emerging evidence indicates that incidence and tumour growth of some cancers associated with the MetS can be attenuated by n-3 PUFA [3]. Independently of the total amount of n-3 PUFA, the n-6/n-3 ratio seems to be determinant as a ratio of 2.5/1 reduced rectal cell proliferation in patients with colorectal cancer, whereas a ratio of 4/1 with the same amount of n-3 PUFA had no effect [77].

## 5. Recommendations for n-3 PUFA Consumption

From the above, it is obvious that there is a need for recommendations for n-3 PUFA nutritional supplies both for the prevention of MetS and associated disorders in the general population and for secondary prevention or treatment. Establishing such guidelines represents a complex issue for three main reasons. First, there is a large interindividual variability in n-3 PUFA metabolism based on genetic determinants, gender and age, further magnified by concurrent associated diseases or epigenetic modifications. Therefore, ideal requirement for physiological effect needs to be tailored for a specific individual [52–54, 78]. Secondly, dietary composition, for example, high dietary SFA or high n-6 PUFA, interferes with the biological effects of n-3 PUFA [31, 77]. The consequences of excessive n-6 PUFA remain controversial: n-6 PUFA have intrinsic cardiovascular protective effects [79], justifying the latest FAO/WHO recommendations on maintaining high n-6 PUFA intakes if n-3 PUFA ones are fulfilled [80]. However, n-6 PUFA compete with n-3 PUFA for processing to eicosanoids, thereby limiting production of antiinflammatory n-3 PUFA derived mediators [46]. Moreover, there are convincing evidence that a low n-6/n-3 PUFA ratio is determinant for the prevention of pathologies associated to the MetS, as colorectal cancer [77] and NAFLD [81–83]. Thus, we propose that n-3 PUFA recommendations must be part of a more global dietary counseling and should be associated with maximum reduction in SFA and limitation of n-6 PUFA intakes to their recommended levels (from 5% to 10% energy intake in Europe and USA [79], resp.). The n-6/n-3 PUFA ratio is a good indicator of this balance. Thirdly, there are concerns about availability of certain foodstuffs (such as wild fish) and food contaminants, as seafood, rich in LC n-3 PUFA, is also a dietary source of heavy metals (methylmercury), polychlorinated biphenyls, dioxins, and other organic pollutants [84]. 

Deduced from ancestral nutrition, in an ideal balanced diet, fat should represent no more than 20%–30% of total energy intake amongst which 5-6 g/d of n-3 PUFA with a great proportion of EPA+DHA and the n-6-to-n-3 ratio should average 1 [17, 30]. To keep in with a developmental approach and with the epigenetic consequences of the diet [56], a ratio of n-6/n-3 around 1 in breast milk should serve as a bench mark to determine the appropriate dietary requirements during pregnancy, lactation, and infant feeding [85].

Previously, health organizations and government agencies in most western countries recommended daily consumption of 0.6 to 1 g n-3 PUFA from which 100–200 mg of LC n-3 PUFA (EPA+DHA). However, in intervention studies reporting a beneficial health effect, the consumption of fish oils or their derivatives resulted in LC n-3 PUFA daily intakes well above those “recommended” 200 mg/day and ranged from 0.5 to 9 g/d. Indeed, in a meta-analysis, a 37% reduction in the relative risk of coronary heart disease in the general population was seen with a daily intake of EPA/DHA of 566 mg. Therefore, this justifies readjustments of nutritional guidelines to an upper level. Governments (France, Belgium, UK, The Netherlands, New Zeeland, and Australia) and health organizations (FAO/WHO, American Dietetic Association, American Heart Association) now recommend dietary intakes for total n-3 PUFA of 1.4 to 2.5 g/d, with EPA and DHA ranging from 140 to 600 mg/d depending on the authority issuing guidelines, FOA/WHO making a relatively low recommendation of 250 mg/d, the average being around 500 mg/d [80, 86, 87]. This represents minimum of 2 servings of fish per week (30–40 g/d), including one of oily fish (salmon, tuna, mackerel, and sardine). In the light of the literature and interindividual variability in PUFA metabolism and requirement, probably the minimal EPA+DHA supplies for healthy adults should reach 0.5–1 g/d (2–4 servings per week of fish, half of oily fish); that is, minimal consumption proved to reduce MetS [86], with a total intake of n-3 PUFA of 5-6 g/d as found in ancestral nutrition to which our metabolism is best fit [18, 51]. Such levels are met in the traditional Japanese diet as it contains 80–100 g fish and shellfish/d/capita [88].

## 6. Are These Recommendations Followed?

To address this question, we calculated FA composition in meals proposed by nutritionist coordinated collective caterings to which health is of concern: first, in lunches supplied by the township of Lille (France) to healthy pupils (4–6 and 6–9 y) and adults and second, in meals proposed to patients hospitalized in St. Luc University Hospital (Brussels, Belgium). 

Total content in FA and specific contents in SFA, MUFA, PUFA, n-6, n-3, and LC n-3 PUFA were calculated in menus over 6 representative weeks for the township collective catering of Lille and in 4 weeks winter menus and 4 weeks summer menus, proposed in rotation along the year by the university hospital. Three types of menus were analyzed: normal, for diabetic patients, and low fat. We used (i) the official table of composition in saturated, mono- and polyunsaturated FA of foodstuffs provided by the French Agency for Food Safety [89], (ii) the table of composition in n-6 and n-3 PUFA of fish, meat, oils, and dairy provided either by the project “Nutritional Composition of Aquatic Products” [27] or by the French Institute for Nutrition [90], and (iii) the EPA and DHA contents of specific foodstuffs provided by the USDA National Nutrient Database for Standard Reference [28]. The ANC guidelines [91] were taken as reference for daily recommended intakes (DRI) and calculated lunch daily recommended intakes (LDRI) as 35%–40% of DRI, with a range representing minimum supplies for girls and maximum ones for boys.

Results are presented in Table 1. In collective lunches proposed by the township of Lille whether to children or adults, the mean contents in FA and SFA were relatively high, estimated at 117%–141% and 116%–149% of LDRI, respectively, and MUFA supplies were relatively insufficient (59%–91%). However, supplies in both total and n-6 PUFA exceeded LDRI by 200%–300%. This is related to systematic replacement of processed fats with safflower oil (rich in n-6) as the main dressing and cooking oil. Strikingly, n-3 PUFA contents were low, representing only 68%–91% of LDRI, although there were 8 servings of fish over the 6 weeks menus, 4 servings were white fish (1.2% fat), 2 canned tuna (4.1% fat), and 2 salmon (11.8% fat), but one of which as small portion served as baked pasta dish. As a result, n-6/n-3 ratio was dramatically elevated (18,6–24,1/1). 

In the meals proposed at St. Luc University Hospital, total FA were relatively low (66%–74% of DRI and 48% of DRI in low fat menus), SFA were in the recommended range or below, but MUFA were dramatically low (38% of DRI and 27% for low fat). Recommended amounts of total and n-6 PUFA were supplied in classical and low-fat diets, but they were outleveled in the diabetic regimen, owing to the addition of 2 safflower-based dressings per day for lunch and evening salads. Regarding n-3 PUFA, the content in total n-3 PUFA was between 1.8 and 1.9 g/d and that of LC n-3 PUFA (EPA+DHA) of 460 mg/day. Those are close to or within the recommendations [91]. Thus, n-6/n-3 ratio varied from 5/1 to 8/1 (the ideal being 1/1, the recommendation 4/1, and currently in the global population 20/1). It is of note that the quasiadequate amounts of n-3 PUFA and LC n-3 PUFA are supplied owing to the presence of 2 portion/d (breakfast, diner) of an n-3 PUFA-enriched margarine containing 16% n-3 PUFA and 0.5% EPA+DHA. This represents 1.2 g/d n-3 PUFA and 0.2 g/d EPA+DHA, without which n-3 PUFA supplies would be insufficient with an n-6/n-3 ratio higher than 15.

Thus, consistent with other reports [17, 29, 30, 77], despite increasing awareness and nutritionist-assisted food catering, reaching adequate or recommended n-3 PUFA supplies in collective nutrition still needs effective and applicable solutions. For reflection, in in-hospital catering, replacement of white fish by fatty fish in one serving has been discussed in order to try to overtake minimum DRI for n-3 PUFA and reduce n-6/n-3 ratio (particularly in menus for diabetic subjects). In school catering, increasing the use of rapeseeds oil (59% MUFA, 20% n-6, and 9% n-3) in replacement of safflower oil (20% MUFA, 64% n-6 and 0.2% n-3) for 50% of the dressings was considered. Forecast calculations show that this would greatly participate in reducing excessive n-6, PUFA intake (−2 g/d) and increase both MUFA (+1,6 g/d) and n-3 PUFA (+0,4 g/d) intakes, resulting in a half reduction of n-6/n-3 ratio (9,5 versus 20). This appears as a simple measure, easily implemented, while very effective. As exemplified in results from hospital menus, the incorporation of enriched manufactured products such as n-3 PUFA-enriched margarine is an alternative to compensate for insufficient supply of natural products.

## 7. How Could We Modify Our Diet to Improve n-3 Intakes?

Fish and oils rich in ALA (flaxseed, canola, soybean, walnut) represent the main sources of n-3 PUFA. As conversion rate from ALA to EPA/DHA is low in humans, a minimum part of the recommended nutritional supplies in n-3 PUFA should be provided as marine LC PUFA (500 mg/d). The first effective measure for increasing n-3 PUFA intakes should consist in actively promoting fish consumption, to reach 35–40 g fish/d. Ideally, wild fish should be given the preference as some species have a higher n-3 PUFA content and/or a lower n-6/n-3 ratio than farmed ones, which usually contain more n-6 PUFA [26–28, 92] partially owing to alimentation. Given the declining stocks of marine fish and high pollution in some fishing areas, this is most likely not a sustainable and globally applicable solution. 

An additional measure is to use ALA rich oils, as envisaged in school catering. For example, replacement of dressing oils rich in n-6 PUFA (mainly safflower oil) by oils rich in n-3 PUFA (flaxseeds, walnuts, wheat germ, rapeseeds, and soybean) provides a substantial additional n-3 PUFA supply (15 g walnuts oil = 1.5 g ALA = 2 dressings) while decreasing n-6 intake. However, such modifications require nutritional education and education to different tastes (oils rich in n-3 PUFA have pronounced tastes), probably easier to integrate onto the developing palette of taste during infancy. As edible wild plants provide higher amounts of ALA and antioxidants than intensively cultivated plants [39], encouraging agricultural methods that are more respectful of developmental cycles and natural nutritional contents of plants would help increase n-3 PUFA consumption. 

Consideration has also to be brought to the cooking methods as n-3 PUFA are highly sensitive to oxidation by oxygen, light, and heat, leading to production of deleterious free radicals. Indeed, cooking fish might reduce by up to 50% its content in n-3 PUFA [93]. Thus consumption of freshly harvested raw (or cooked at low heat) fish and raw n-3 PUFA-rich oils should be promoted. 

Food enrichment is emerging as perhaps the best long-term solution to the chronically low intake of n-3 PUFA that plagues western cultures [87]. First, n-3 PUFA-rich oils should more systematically replace n-6 PUFA-rich oils in industrial preparations. Second, efforts are being made to produce a variety of food products, most notably eggs, yogurt, milk, and spreads, enriched with n-3 PUFA-rich food [94]. Alternatively, n-3 PUFA synthesis might be induced by genetic manipulation. This has been done in plants by transgene-driven expression of Δ6 desaturase. In derived oil, LC n-3 PUFA concentration amounts those found in native marine organisms [95]. At the experimental level, this application has been extended to mice. Indeed, the team of Kang realized a stable transfection of FADS3 from *C. Elegans*, an enzyme missing in mammals which catalyses conversion of n-6 to n-3 PUFA [96]. This resulted in spontaneous enrichment of their lipids with n-3 PUFA. Such experiment might pave the way to genetic manipulation of cattle and poultry to produce n-3 PUFA-rich raw material. Innumerable ethical, ecological, economical, and cultural issues need to be addressed prior to generalization of such experimental trials.

## 8. Concluding Remarks

The literature provides compelling evidence for the health benefit of n-3 PUFA consumption not only on the MetS, cardiovascular risks, and associated comorbidities but also on other conditions such as neuroinflammatory and neurodegenerative diseases. This evidence must be taken into consideration, and efforts have to be made to promote increased n-3 PUFA consumption together with lowering intakes of high glycemic food, fructose, and fat, in particular SFA and n-6 PUFA when clearly excessive, and increasing ingestion of fruits, vegetables, whole grains, and nuts. Despite this awareness, what is still needed is an educational program for professionals and for the public [17] as well as manifestation of the willingness of governments to institute changes, notably through achieving accountable decisions on catering (as undertaken here) as well as modifications of food processing and industrial food ingredients legislations, all towards promoted use and preservation of n-3 PUFA rich food. Increasing experimental evidence supporting the pivotal roles of nutrition in the regulation of homeostasis highlight our neglected responsibility in promoting balanced diet and consumption of food rich in essential nutrients in the general population.

## Figures and Tables

**Table 1:** Daily fatty acids supplies in municipal and hospital catering.

		FA			
		Total g (% LDRI)	SFA g (% LDRI)	MUFA g (% LDRI)	PUFA g (% LDRI)
Lille municipal catering	Children 4*–*6 y	28,3 (141%)	8,5 (139%)	8,4 (91%)	10,3 (381%)
	*LDRI *min-max	*16,5*–*23,8 *	^ a^ *5,5*–*6,8 *	*6,6*–*11,9 *	*1,3*–*4,1 *
	Children 6*–*10 y	30,0 (117%)	9,1 (116%)	9,0 (76%)	10,7 (310%)
	*LDRI *min-max	*20,9*–*30,2 *	^ a^ *7,0*–*8,6 *	*8,4*–*15,1 *	*1,7*–*5,2 *
	Adults	32,6 (117%)	10,0 (149%)	9,9 (59%)	11,3 (265%)
	*LDRI *min-max	*23,1*–*32,4 *	^ a^ *5,6*–*7,8 *	*14,0*–*19,6 *	*3,5*–*5,0 *

St. Luc Hospital, Brussels	Classic	48,28 (66%)	18,38 (104%)	16,79 (38%)	13,10 (116%)
					^ b^11,90 (105%)
	Diabetic	54,15 (74%)	20,16 (114%)	17,52 (39%)	16,47 (146%)
					^ b^15,27 (135%)
	Low-fat	35,36 (48%)	12,64 (71%)	12,07 (27%)	10,64 (95%)
					^ b^9,44 (84%)
	*DRI women-men*	*66*–*81 *	^ a^ *16,0*–*19,5 *	*40,0*–*49,0 *	*10,0*–*12,5 *

		PUFA			
		n-6 PUFA g (% DRI)	n-3 PUFA g (% DRI)	EPA+DHA g (% DRI)	n-6/n-3 ratio (% DRI)

Lille municipal catering	Children 4*–*6 y	9,9 (441%)	0,4 (91%)	0,16	24,1 (482%)
	*LDRI *min-max	*1,1*–*3,4 *	*0,2*–*0,7 *		*5,00*
	Children 6*–*10 y	10,2 (359%)	0,5 (82%)	0,22	20,8 (415%)
	*LDRI *min-max	*1,4*–*4,3 *	*0,3*–*0,9 *		*5,00*
	Adults	10,8 (316%)	0,6 (68%)	0,27 (130%)	18,6 (466%)
	*LDRI *min-max	*2,8*–*4,0 *	*0,7*–*1,0 *	*0,20*	*4,00*

St. Luc Hospital, Brussels	Classic	11,24 (125%)	1,92 (85%)	0,46 (93%)	5,86 (147%)
			^ b^0,72 (32%)	^ b^0,26 (53%)	^ b^15,61 (390%)
	Diabetic	14,66 (163%)	1,85 (82%)	0,46 (92%)	7,91 (198%)
			^ b^0,65 (29%)	^ b^0,26 (52%)	^ b^22,55 (563%)
	Low-fat	8,90 (99%)	1,79 (80%)	0,46 (93%)	4,96 (124%)
			^ b^0,59 (26%)	^ b^0,26 (53%)	^ b^15,08 (377%)
	*DRI women-men*	*8,0*–*10,0 *	*2,0*–*2,5 *	*0,50*	*4,00*

LDRI: lunch daily recommended intakes [91]; numbers in brackets correspond to the % of average DRI/LDRI; ^a^For SFA, values represent the range of 35% of the maximum intakes for women and 40% of the maximum intakes for men; ^b^Values without enriched margarine.

## Abbreviations

ALA: Alpha-linolenic acid
COX: Cylooxygenase
DHA: Docosahexaenoic acid
DRI: Dietary recommended intake
EPA: Eicosapentaenoic acid
FA: Fatty acids
FAD: Fatty acid desaturase
LC PUFA: Long-chain polyunsaturated fatty acids
LDRI: Lunch dietary recommended intake
LOX: Lipoxygenase
LT: Leukotriene
MetS: Metabolic syndrome
MUFA: Monounsaturated fatty acids
NAFLD: Nonalcoholic fatty liver disease
PG: Prostaglandin
PUFA: Polyunsaturated fatty acids
T2DM: Type 2 diabetes mellitus.

## References

[1] Hwu C-M, Hsiung CA, Wu K-D (2008). Diagnosis of insulin resistance in hypertensive patients by the metabolic syndrome: AHA vs. IDF definitions. *International Journal of Clinical Practice*.

[2] Byrne CD (2010). Fatty liver: role of inflammation and fatty acid nutrition. *Prostaglandins Leukotrienes and Essential Fatty Acids*.

[3] Cerchietti LCA, Navigante AH, Castro MA (2007). Effects of eicosapentaenoic and docosahexaenoic n-3 fatty acids from fish oil and preferential Cox-2 inhibition on systemic syndromes in patients with advanced lung cancer. *Nutrition and Cancer*.

[4] Giugliano D, Ceriello A, Esposito K (2008). Are there specific treatments for the metabolic syndrome?. *American Journal of Clinical Nutrition*.

[5] Das UN (2002). Is metabolic syndrome X an inflammatory condition?. *Experimental Biology and Medicine*.

[6] Das UN (2002). Obesity, metabolic syndrome X, and inflammation. *Nutrition*.

[7] Bulló M, Casas-Agustench P, Amigó-Correig P, Aranceta J, Salas-Salvadó J (2007). Inflammation, obesity and comorbidities: the role of diet. *Public Health Nutrition*.

[8] Ferrante AW (2007). Obesity-induced inflammation: a metabolic dialogue in the language of inflammation. *Journal of Internal Medicine*.

[9] Lanthier N, Molendi-Coste O, Horsmans Y, Van Rooijen N, Cani PD, Leclercq IA (2010). Kupffer cell activation is a causal factor for hepatic insulin resistance. *American Journal of Physiology*.

[10] Luczynski W, Bossowski A, Glowinska-Olszewska B, Kos J, Stasiak-Barmuta A (2010). The role of T-regulatory cells in the pathogenesis of immunological disturbances accompanying obesity and atherosclerosis. *Postępy Higieny i Medycyny Doświadczalne*.

[11] Life Sciences Research Office FoASfEB (1995). *Third Report on Nutrition Monitoring in the United States*.

[12] Cordain L, Eaton SB, Sebastian A (2005). Origins and evolution of the Western diet: health implications for the 21st century. *American Journal of Clinical Nutrition*.

[13] Schmidhuber J The EU Diet–Evolution, Evaluation and Impacts of the CAP. Document presented at the WHO Forum on Trade and Healthy Food and Diets Montréal.

[14] Ferder L, Ferder MD, Inserra F (2010). The role of high-fructose corn syrup in metabolic syndrome and hypertension. *Current Hypertension Reports*.

[15] Linseisen J, Welch AA, Ocke M (2009). Dietary fat intake in the European prospective investigation into cancer and nutrition: results from the 24-h dietary recalls. *European Journal of Clinical Nutrition*.

[16] Lupien JR, Richmond A, Randell M, Cotier JP, Ghazali A, Dawson R (1994). *Food, Nutrition and Agriculture. Edible Fats and Oils*.

[17] Simopoulos AP (2001). n-3 fatty acids and human health: defining strategies for public policy. *Lipids*.

[18] Eaton SB, Konner MJ, Cordain L (2010). Diet-dependent acid load, Paleolithic nutrition, and evolutionary health promotion. *American Journal of Clinical Nutrition*.

[19] Simopoulos AP (1994). Is insulin resistance influenced by dietary linoleic acid and trans fatty acids?. *Free Radical Biology and Medicine*.

[20] Napier JA, Sayanova O, Qi B, Lazarus CM (2004). Progress toward the production of long-chain polyunsaturated fatty acids in transgenic plants. *Lipids*.

[21] Ruidavets J-B, Bongard V, Dallongeville J (2007). High consumptions of grain, fish, dairy products and combinations of these are associated with a low prevalence of metabolic syndrome. *Journal of Epidemiology and Community Health*.

[22] Ruiz-López N, Haslam RP, Venegas-Calerón M (2009). The synthesis and accumulation of stearidonic acid in transgenic plants: a novel source of ’heart-healthy’ omega-3 fatty acids. *Plant Biotechnology Journal*.

[23] Flachs P, Rossmeisl M, Bryhn M, Kopecky J (2009). Cellular and molecular effects of n-3 polyunsaturated fatty acids on adipose tissue biology and metabolism. *Clinical Science*.

[24] Kris-Etherton PM, Taylor DS, Yu-Poth S (2000). Polyunsaturated fatty acids in the food chain in the United States. *American Journal of Clinical Nutrition*.

[25] Welch AA, Lund E, Amiano P (2002). Variability of fish consumption within the 10 European countries participating in the European Investigation into Cancer and Nutrition (EPIC) study. *Public Health Nutrition*.

[26] Blanchet C, Lucas M, Julien P, Morin R, Gingras S, Dewailly É (2005). Fatty acid composition of wild and farmed Atlantic salmon (*Salmo salar*) and rainbow trout (*Oncorhynchus mykiss*). *Lipids*.

[27] Pôle "Filière Produits Aquatiques" ADRIA Normandie, CEVPM, ID Mer, ITERG, ISHA. Projet Composition nutritionnelle des produits aquatiques. www.nutraqua.com.

[28] U.S. Department of Agriculture ARS USDA National Nutrient Database for Standard Reference, Release 22.

[29] Anderson BM, Ma DWL (2009). Are all n-3 polyunsaturated fatty acids created equal?. *Lipids in Health and Disease*.

[30] Sanders TAB (2000). Polyunsaturated fatty acids in the food chain in Europe. *American Journal of Clinical Nutrition*.

[31] Ebbesson SO, Tejero ME, Nobmann ED (2007). Fatty acid consumption and metabolic syndrome components: the GOCADAN study. *Journal of the Cardiometabolic Syndrome*.

[32] Kennedy A, Martinez K, Chuang C-C, Lapoint K, Mcintosh M (2009). Saturated fatty acid-mediated inflammation and insulin resistance in adipose tissue: mechanisms of action and implications. *Journal of Nutrition*.

[33] Dolecek TA (1992). Epidemiological evidence of relationships between dietary polyunsaturated fatty acids and mortality in the Multiple Risk Factor Intervention Trial. *Proceedings of the Society for Experimental Biology and Medicine*.

[34] Delavar MA, Lye MS, Khor GL, Hassan ST, Hanachi P (2009). Dietary patterns and the metabolic syndrome in middle aged women, Babol, Iran. *Asia Pacific Journal of Clinical Nutrition*.

[35] Zuliani G, Galvani M, Leitersdorf E, Volpato S, Cavalieri M, Fellin R (2009). The role of polyunsaturated fatty acids (PUFA) in the treatment of dyslipidemias. *Current Pharmaceutical Design*.

[36] Das UN (2008). Essential fatty acids and their metabolites could function as endogenous HMG-CoA reductase and ACE enzyme inhibitors, anti-arrhythmic, anti-hypertensive, anti-atherosclerotic, anti-inflammatory, cytoprotective, and cardioprotective molecules. *Lipids in Health and Disease*.

[37] Harris WS, Mozaffarian D, Lefevre M (2009). Towards establishing dietary reference intakes for eicosapentaenoic and docosahexaenoic acids. *Journal of Nutrition*.

[38] Ruxton CHS, Reed SC, Simpson JA, Millington KJ (2007). The health benefits of omega-3 polyunsaturated fatty acids: a review of the evidence. *Journal of Human Nutrition and Dietetics*.

[39] Simopoulos AP (2004). Omega-3 fatty acids and antioxidants in edible wild plants. *Biological Research*.

[40] Ferrucci L, Cherubini A, Bandinelli S (2006). Relationship of plasma polyunsaturated fatty acids to circulating inflammatory markers. *Journal of Clinical Endocrinology and Metabolism*.

[41] Kopecky J, Rossmeisl M, Flachs P (2009). n-3 PUFA: bioavailability and modulation of adipose tissue function. *Proceedings of the Nutrition Society*.

[42] Lattka E, Illig T, Heinrich J, Koletzko B (2009). Do *FADS* genotypes enhance our knowledge about fatty acid related phenotypes?. *Clinical Nutrition*.

[43] Serhan CN (2009). Systems approach to inflammation resolution: identification of novel anti-inflammatory and pro-resolving mediators. *Journal of Thrombosis and Haemostasis*.

[44] Das UN (2005). A defect in the activity of Δ6 and Δ5 desaturases may be a factor predisposing to the development of insulin resistance syndrome. *Prostaglandins Leukotrienes and Essential Fatty Acids*.

[45] Devlin AM, Singh R, Wade RE, Innis SM, Bottiglieri T, Lentz SR (2007). Hypermethylation of Fads2 and altered hepatic fatty acid and phospholipid metabolism in mice with hyperhomocysteinemia. *Journal of Biological Chemistry*.

[46] Bannenberg GL (2010). Therapeutic applicability of anti-inflammatory and proresolving polyunsaturated fatty acid-derived lipid mediators. *The Scientific World Journal*.

[47] Lagarde M, Chen P, Véricel E, Guichardant M (2010). Fatty acid-derived lipid mediators and blood platelet aggregation. *Prostaglandins Leukotrienes and Essential Fatty Acids*.

[48] Das UN (2006). Essential fatty acids—a review. *Current Pharmaceutical Biotechnology*.

[49] Visioli F, Hagen TM (2007). Nutritional strategies for healthy cardiovascular aging: focus on micronutrients. *Pharmacological Research*.

[50] Farzaneh-Far R, Lin J, Epel ES, Harris WS, Blackburn EH, Whooley MA (2010). Association of marine omega-3 fatty acid levels with telomeric aging in patients with coronary heart disease. *Journal of the American Medical Association*.

[51] Simopoulos AP (2006). Evolutionary aspects ofdiet, theomega-6/omega-3ratio andgenetic variation: nutritional implications forchronic diseases. *Biomedicine and Pharmacotherapy*.

[52] Lattka E, Illig T, Koletzko B, Heinrich J (2010). Genetic variants of the FADS1 FADS2 gene cluster as related to essential fatty acid metabolism. *Current Opinion in Lipidology*.

[53] Allayee H, Roth N, Hodis HN (2009). Polyunsaturated fatty acids and cardiovascular disease: implications for nutrigenetics. *Journal of Nutrigenetics and Nutrigenomics*.

[54] Reese AC, Fradet V, Witte JS (2009). *ω*-3 Fatty acids, genetic variants in COX-2 and prostate cancer. *Journal of Nutrigenetics and Nutrigenomics*.

[55] Das UN (2007). Is metabolic syndrome X a disorder of the brain with the initiation of low-grade systemic inflammatory events during the perinatal period?. *Journal of Nutritional Biochemistry*.

[56] Chmurzynska A (2010). Fetal programming: link between early nutrition, DNA methylation, and complex diseases. *Nutrition Reviews*.

[57] Lopez-Garcia E, Schulze MB, Manson JE (2004). Consumption of (n-3) fatty acids is related to plasma biomarkers of inflammation and endothelial activation in women. *Journal of Nutrition*.

[58] Das UN (1999). GLUT-4, tumour necrosis factor, essential fatty acids and daf-genes and their role in glucose homeostasis, insulin resistance, non-insulin dependent diabetes mellitus, and longevity. *Journal of Association of Physicians of India*.

[59] Madsen T, Skou HA, Hansen VE (2001). C-reactive protein, dietary n-3 fatty acids, and the extent of coronary artery disease. *American Journal of Cardiology*.

[60] Das UN (2005). Long-chain polyunsaturated fatty acids, endothelial lipase and atherosclerosis. *Prostaglandins Leukotrienes and Essential Fatty Acids*.

[61] Chan DC, Watts GF, Barrett PHR, Beilin LJ, Mori TA (2002). Effect of atorvastatin and fish oil on plasma high-sensitivity C-reactive protein concentrations in individuals with visceral obesity. *Clinical Chemistry*.

[62] Mori TA, Woodman RJ, Burke V, Puddey IB, Croft KD, Beilin LJ (2003). Effect of eicosapentaenoic acid and docosahexaenoic acid on oxidative stress and inflammatory markers in treated-hypertensive type 2 diabetic subjects. *Free Radical Biology and Medicine*.

[63] Rallidis LS, Paschos G, Liakos GK, Velissaridou AH, Anastasiadis G, Zampelas A (2003). Dietary *α*-linolenic acid decreases C-reactive protein, serum amyloid A and interleukin-6 in dyslipidaemic patients. *Atherosclerosis*.

[64] Zhao G, Etherton TD, Martin KR, West SG, Gillies PJ, Kris-Etherton PM (2004). Dietary *α*-linolenic acid reduces inflammatory and lipid cardiovascular risk factors in hypercholesterolemic men and women. *Journal of Nutrition*.

[65] Couet C, Delarue J, Ritz P, Antoine J-M, Lamisse F (1997). Effect of dietary fish oil on body fat mass and basal fat oxidation in healthy adults. *International Journal of Obesity*.

[66] Abete I, Astrup A, Martínez JA, Thorsdottir I, Zulet MA (2010). Obesity and the metabolic syndrome: role of different dietary macronutrient distribution patterns and specific nutritional components on weight loss and maintenance. *Nutrition Reviews*.

[67] Todoric J, Löffler M, Huber J (2006). Adipose tissue inflammation induced by high-fat diet in obese diabetic mice is prevented by n-3 polyunsaturated fatty acids. *Diabetologia*.

[68] Mattar M, Obeid O (2009). Fish oil and the management of hypertriglyceridemia. *Nutrition and Health*.

[69] De Santa Olalla LM, Sáchez Muniz FJ, Vaquero MP (2009). N-3 fatty acids in glucose metabolism and insulin sensitivity. *Nutricion Hospitalaria*.

[70] Peyron-Caso E, Fluteau-Nadler S, Kabir M (2002). Regulation of glucose transport and transporter 4 (Glut-4) in muscle and adipocytes of sucrose-fed rats: effects of n-3 poly- and monounsaturated fatty acids. *Hormone and Metabolic Research*.

[71] Ramel AL, Jonsdottir MT, Thorsdottir I (2009). Consumption of cod and weight loss in young overweight and obese adults on an energy reduced diet for 8-weeks. *Nutrition, Metabolism and Cardiovascular Diseases*.

[72] Ebrahimi M, Ghayour-Mobarhan M, Rezaiean S (2009). Omega-3 fatty acid supplements improve the cardiovascular risk profile of subjects with metabolic syndrome, including markers of inflammation and auto-immunity. *Acta Cardiologica*.

[73] Mori TA, Bao DQ, Burke V, Puddey IB, Beilin LJ (1999). Docosahexaenoic acid but not eicosapentaenoic acid lowers ambulatory blood pressure and heart rate in humans. *Hypertension*.

[74] Reiner Ž, Tedeschi-Reiner E, Štajminger G (2007). The role of omega-3 fatty acids from fish in prevention of cardiovascular diseases. *Lijecnicki Vjesnik*.

[75] Rapoport SI, Igarashi M, Gao F (2010). Quantitative contributions of diet and liver synthesis to docosahexaenoic acid homeostasis. *Prostaglandins Leukotrienes and Essential Fatty Acids*.

[76] Bozza PT, Viola JPB (2010). Lipid droplets in inflammation and cancer. *Prostaglandins Leukotrienes and Essential Fatty Acids*.

[77] Simopoulos AP (2002). The importance of the ratio of omega-6/omega-3 essential fatty acids. *Biomedicine and Pharmacotherapy*.

[78] Simopoulos AP (2010). Nutrigenetics/nutrigenomics. *Annual Review of Public Health*.

[79] Harris WS, Mozaffarian D, Rimm E (2009). Omega-6 fatty acids and risk for cardiovascular disease: a science advisory from the American Heart Association nutrition subcommittee of the council on nutrition, physical activity, and metabolism; council on cardiovascular nursing; and council on epidemiology and prevention. *Circulation*.

[81] Cortez-Pinto H, Jesus L, Barros H, Lopes C, Moura MC, Camilo ME (2006). How different is the dietary pattern in non-alcoholic steatohepatitis patients?. *Clinical Nutrition*.

[82] Musso G, Gambino R, De Michieli F (2003). Dietary habits and their relations to insulin resistance and postprandial lipemia in nonalcoholic steatohepatitis. *Hepatology*.

[83] Vuppalanchi R, Cummings OW, Saxena R (2007). Relationship among histologic, radiologic, and biochemical assessments of hepatic steatosis: a study of human liver samples. *Journal of Clinical Gastroenterology*.

[84] Domingo JL, Bocio A (2007). Levels of PCDD/PCDFs and PCBs in edible marine species and human intake: a literature review. *Environment International*.

[85] Simopoulos AP (1991). Omega-3 fatty acids in health and disease and in growth and development. *American Journal of Clinical Nutrition*.

[86] Yashodhara BM, Umakanth S, Pappachan JM, Bhat SK, Kamath R, Choo BH (2009). Omega-3 fatty acids: a comprehensive review of their role in health and disease. *Postgraduate Medical Journal*.

[87] Harris WS (2007). International recommendations for consumption of long-chain omega-3 fatty acids. *Journal of Cardiovascular Medicine*.

[88] Nagata C, Takatsuka N, Shimizu H (2002). Soy and fish oil intake and mortality in a Japanese community. *American Journal of Epidemiology*.

[89] French Agency for Food Safety (AFSSA) Table de composition nutritionnelle des aliments Ciqual 2008. http://www.afssa.fr/TableCIQUAL/.

[90] Institut Français pour la Nutrition (2003). *Dossier Scientifique sur les Lipides*.

[91] (2000). *Centre national d'études et de recommandations sur la nutrition et l'alimentation C, Centre national de la recherche scientifique C*.

[92] Kermouni-Giorgio B, Ollivier D, Marescot H Différenciation entre poisson sauvage et poisson d'élevage.

[93] Moradi Y, Bakar J, Syed Muhamad SH, Che Man Y (2009). Effects of different final cooking methods on physico-chemical properties of breaded fish fillets. *American Journal of Food Technology*.

[94] Riediger ND, Othman RA, Suh M, Moghadasian MH (2009). A systemic review of the roles of n-3 fatty acids in health and disease. *Journal of the American Dietetic Association*.

[95] Napier JA, Graham IA (2010). Tailoring plant lipid composition: designer oilseeds come of age. *Current Opinion in Plant Biology*.

[96] Kang JX, Watson RR, DeMeester F (2007). Omega-6/Omega-3 fatty acid ratio is important for health. Lessons from genetically modified cells and animals. *Wild-Type Food in Health Promotion and Disease Prevention*.

